# Quality of Life, Risk and Recovery in a National Forensic Mental Health Service: A D-FOREST study from DUNDRUM Hospital

**DOI:** 10.1192/j.eurpsy.2022.1545

**Published:** 2022-09-01

**Authors:** H. Amin, I. Edet, N. Basrak, G. Crudden, H. Kennedy, M. Davoren

**Affiliations:** 1 National Forensic Mental Health Service, Ireland, Research, Dublin, Ireland; 2 Health Service Executive, Psychiatry, Dublin, Ireland; 3 Health Service Executive, Psychiatry, Galway, Ireland; 4 Central Mental Hospital and Trinity College Dublin, Dundrum Centre For Forensic Excellence, Dublin, Ireland; 5 Department of Psychiatry, Trinity College Dublin, Dublin, Ireland; 6 National Forensic Mental Health Service, Central Mental Hospital, Dundrum, Dublin, Ireland; 7 Central Mental Hospital, National Forensic Mental Health Service, Dundrum, Dublin, Ireland

**Keywords:** Forensic in-patients, Quality of Life, Risk, Recovery

## Abstract

**Introduction:**

Secure forensic mental health services have a dual role, to treat mental disorder and reduce violent recidivism. Quality of life is a method of assessing an individual patients’ perception of their own life and is linked to personal recovery. Placement in secure forensic hospital settings should not be a barrier to achieving meaningful quality of life. The WHO-QuOL measure is a self-rated tool, internationally validated used to measure patients own perception of their quality of life.

**Objectives:**

This aim of this study was to assess self-reported quality of life in a complete National cohort of forensic in-patients, and ascertain the associations between quality of life and measures of violence risk, recovery and functioning.

**Methods:**

This is a cross sectional study, set in Dundrum Hospital, the site of Ireland’s National Forensic Mental Health Service. It therefore includes a complete national cohort of forensic in-patients. The WHO-QuOL was offered to all 95 in-patients in Dundrum Hospital during December 2020 – January 2021, as was PANSS (Positive and Negative Symptoms for Schizophrenia Scale). During the study period the researchers collated the scores from HCR-20 (violence risk), therapeutic programme completion (DUNDRUM-3) and recovery (DUNDRUM-4). Data was gathered as part of the Dundrum Forensic Redevelopment Evaluation Study (D-FOREST).

**Results:**

Lower scores on dynamic violence risk, better recovery and functioning scores were associated with higher self-rated quality of life.

**Conclusions:**

The quality of life scale was meaningful in a secure forensic hospital setting. Further analysis will test relationships between symptoms, risk and protective factors and global function.

**Disclosure:**

No significant relationships.

